# Supplementary material for: The Transition to Noncommunicable Disease: How to Reduce Its Unsustainable Global Burden by Increasing Cognitive Access to Health Self-Management
Source: J Intell. 2021 Dec 9;9(4):61. doi: 10.3390/jintelligence9040061 (PMC8705641; doi:10.3390/jintelligence9040061)
Supplement: Supplementary file 1 [file jintelligence-09-00061-s001.zip › jintelligence-1346761-supplementary.pdf]

Table S1. Sample task analysis of label use that illustrates how to uncover a task's nonobvious demands for information processing. (From "Safe-Guarding Cognitive Access to Diabetes Self-Management as Abilities Decline With Age" by Gottfredson, Linda S., and Kathy Stroh. 2021, *The Diabetes Communicator, Spring 2021,* 9–11. Copyright 2021 by Canadian Diabetes Association).

<table>
<tr><th colspan="2">Self-management task</th></tr>
<tr><td>Here is the nutrition label for your snack later today.

You have diabetes, so you want to eat the right amount of carbohydrate. Assume for now that it is 15 grams (g) for a snack.

Use this label to figure out how much of the product you should eat to get that amount.</td><td>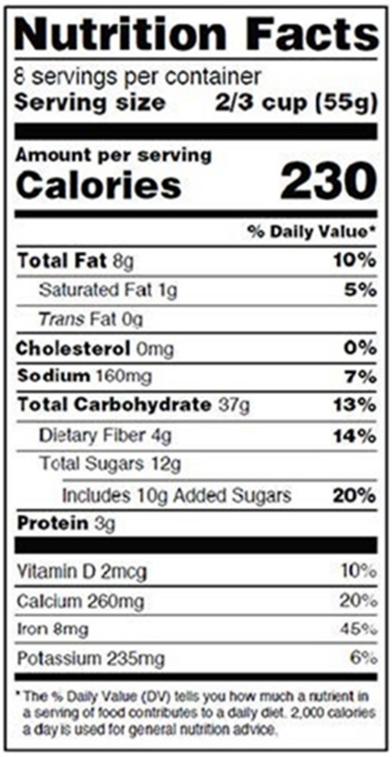
</td></tr>
<tr><th colspan="2">A. Steps in this task</th></tr>
<tr><td colspan="2">1. How much carbohydrate is in one serving?</td></tr>
<tr><td colspan="2">a. Recall that you need to locate an entry for carbohydrate.</td></tr>
<tr><td colspan="2">b. Cycle through the information on the label to find the correct entry for carbohydrate: total carbohydrate.</td></tr>
<tr><td colspan="2">c. Ignore the entries for total sugar and added sugar. These sources of carbohydrate are included in total carbohydrate, as indicated by their indentation.</td></tr>
<tr><td colspan="2">d. Ignore the entry for calories. It is not relevant to your task.</td></tr>
<tr><td colspan="2">e. Ignore all the other nutrients, whether healthy for you (protein, vitamins and minerals) or not (fat, cholesterol, sodium). They are not relevant to your task.</td></tr>
<tr><td colspan="2">f. Recall that carbohydrate is measured in g.</td></tr>
<tr><td colspan="2">g. Locate the number of grams listed for total carbohydrate (here, 37g).</td></tr>
<tr><td colspan="2">h. Ignore the other number after total carbohydrate – 13 – for % daily value. It is not relevant.</td></tr>
<tr><td colspan="2">i. Recall that 37g of total carbohydrate is for only one serving and that the package may contain more than one serving.</td></tr>
<tr><td colspan="2">2. How much of the product in the package counts as one serving?</td></tr>
<tr><td colspan="2">a. Understand that one serving is a standard amount, not the amount that you might think of as a serving.</td></tr>
<tr><td colspan="2">b. Cycle again through the array of information to locate entries for number or size of servings in the package.</td></tr>
<tr><td colspan="2">c. Recognize that two entries near the top are relevant: 8 servings per container and serving size 2/3 cup (55g).</td></tr>
<tr><td colspan="2">d. Understand that in the entry for serving size, 2/3 means two-thirds and that a cup refers to a standard measuring cup, neither a teacup nor a mug.</td></tr>
</table>

e. Understand that the parentheses – ( ) – around 55g for serving size means that this quantity provides a second way to measure one serving.
f. Understand that this second way of measuring serving size, 55g, is for the total weight of one serving, not the g (grams) of total carbohydrate in it (here, 37).
**3. How much of the package will give you the right amount of carbohydrate?**
a. Recall that 15g is the amount of carbohydrate allowed for a snack.
b. Determine whether one serving gives you the right amount, too much or too little carbohydrate. (In this case it is too much because 37g is more than twice 15, so you can't eat more than half a serving.)
c. Select the correct numbers to calculate how much of the product constitutes half a serving. There are three options: number of servings (8), serving size by volume (2/3 cup) and serving size by weight (55g).
d. If you opt to use number of servings (8), select the appropriate arithmetic operation(s) to calculate how much of the package you can eat. If one snack is roughly half a serving, then one serving equals two snacks. Multiply 2 (snacks in a serving) by 8 (number of servings) to get the number of snacks in the packet: 16. Divide the packet into 16 parts and take one or estimate in some other way how much to take out for 1/16 of the package.
e. If you opt instead to use serving size 2/3 cup, select the appropriate arithmetic operation(s) to calculate how much of the packet you can eat. If one snack is half a serving, divide 2/3 cup (one serving) by 2 (number of snacks in a serving) to get half a serving (1/3 cup). Use a standard measuring cup to remove 1/3 cup of the packet's contents.
f. If you opt to use serving size 55g, select the appropriate arithmetic operation(s) to calculate how much of the packet you can eat. If one snack is about half that by weight, use a kitchen scale to take about 27g out of the package. Since 55g of snack contains 37g carbohydrate, you will be taking out half a serving (which will be about 18g carbohydrate).
**B. Elements of information processing that add to a task's complexity (the number-letter combinations refer to steps above)**
1abf, 3a. Requires technical knowledge specific to diabetes: nutrient(s) that affect blood glucose level, that their amounts are measured in number of grams and the amount recommended for a snack.
1c, 2adef. Requires technical knowledge not specific to diabetes: abbreviations (g, mg, mcg), mathematical symbols (%, /), writing conventions (subcategories are indented) and measurement conventions (cup, serving size).
1bcde. Nutrient entries are for abstract categories (fat, carbohydrate, sugars).
1c. Some nutrient entries are subcategories of others: dietary fiber and total sugar, of total carbohydrate.
1cdh. The label contains irrelevant entries that resemble or are adjacent to relevant ones.
1b, 2b. Cycle twice through an array of information to locate two different and nonadjacent types of information.
3a. Label does not provide all the information required to calculate recommended amount of snack to eat.
3c. Select appropriate data for arithmetic calculations.
3bdef. Select appropriate arithmetic operation(s) to calculate an answer.
3def. Choice of arithmetic operation(s) is contingent on type of data chosen to calculate the answer: number, weight or volume of one serving.
3bdef. The arithmetic operations require multiplication or division, which are harder than addition or subtraction.
3e. The arithmetic operations require manipulating fractions or decimals, which is harder than whole numbers.

Source of label: U.S. Food and Drug Administration. Changes to the Nutrition Facts Label. Available at: www.fda.gov/Food/GuidanceRegulation/GuidanceDocumentsRegulatoryInformation/LabelingNutrition/ucm385663.htm (accessed on 7 March 2021).

Table S2. Example of instructor using Bloom's taxonomy of educational objectives (cognitive domain) to sequence instruction by complexity of information processing. (From "Safe-Guarding Cognitive Access to Diabetes Self-Management as Abilities Decline With Age" by Gottfredson, Linda S., and Kathy Stroh. 2021, *The Diabetes Communicator, Spring 2021,* 9–11. Copyright 2021 by Canadian Diabetes Association)

<table>
<tr><td colspan="3">Instructor's task: Use this label to help an individual master using nutrition labels to plan a meal (breakfast) with the recommended amount of carbohydrate.</td></tr>
<tr><td>Nutrition label</td><td>Action verbs</td><td>Objectives: individual must show mastery of each</td></tr>
<tr><td rowspan="6">Nutrition Facts / Valeur nutritive
Serving Size 1 cup / Portion 1 tasse
Servings Per Container 10 / Portions par contenant 10
<table>
<tr><th>Amount
Teneur</th><th>Cereal
Céréale</th><th>Cereal w/milk†
Céréales avec du lait†</th></tr>
<tr><td>Calories / Calories</td><td>190</td><td>250</td></tr>
<tr><td></td><td colspan="2">% Daily Value / % valeur quotidienne</td></tr>
<tr><td>Fat / Lipides 4 g*</td><td>6 %</td><td>8 %</td></tr>
<tr><td>Saturated / saturés 1.5 g
+ Trans / trans 0 g</td><td>8 %</td><td>13 %</td></tr>
<tr><td>Cholesterol / Cholestérol 0 mg</td><td></td><td></td></tr>
<tr><td>Sodium / Sodium 190 mg</td><td>8 %</td><td>11 %</td></tr>
<tr><td>Carbohydrate / Glucides 39 g</td><td>13 %</td><td>15 %</td></tr>
<tr><td>Fibre / Fibres 6 g</td><td>24 %</td><td>24 %</td></tr>
<tr><td>Sugars / Sucres 11 g</td><td></td><td></td></tr>
<tr><td>Protein / Protéines 4 g</td><td></td><td></td></tr>
<tr><td>Vitamin A / Vitamine A</td><td>0 %</td><td>6 %</td></tr>
<tr><td>Vitamin C / Vitamine C</td><td>0 %</td><td>2 %</td></tr>
<tr><td>Calcium / Calcium</td><td>2 %</td><td>15 %</td></tr>
<tr><td>Iron / Fer</td><td>8 %</td><td>8 %</td></tr>
</table>
* Amount in Cereal / Teneur de Céréale
† Cereal w/milk adds 60 Calories, 1 g Fat (1 g Saturated), 10 mg Cholesterol, 70 mg Sodium, 7 g Carbohydrate (6 g Sugars), 5 g Protein. / Céréales avec du lait ajoute 60 Calories, 1 g Lipides (1 g saturés), 10 mg Cholestérol, 70 mg Sodium, 7 g Glucides (6 g Sucres), 5 g Protéines.</td><td colspan="2">1. Remember</td></tr>
<tr><td>Recognize, recall, identify</td><td>State the amount of carbohydrate recommended for your breakfast (in grams).
Name all ingredients on the label that contain carbohydrate (cereal, milk).
If adding other items to meal, name the ones that do (fruit) and do not contain carbohydrate (black coffee).
Identify foods that might not have nutrition labels.</td></tr>
<tr><td colspan="2">2. Understand</td></tr>
<tr><td>Paraphrase, summarize, compare, infer</td><td>Explain why the entries for carbohydrate and serving size are important.
Infer that this label's entry for carbohydrate does not include the carbohydrate in milk.
Explain how to find the carbohydrate content of unlabeled foods.</td></tr>
<tr><td colspan="2">3. Apply</td></tr>
<tr><td>Apply a procedure to carry out a task</td><td>Locate all entries on the label relevant to counting carbohydrate:
• Serving size (1 cup);
• Servings per container (10);
• Carbohydrate for cereal (39g);
• Carbohydrate for milk (7g, in footnote†).
Calculate carbohydrate in foods added to the meal.
Calculate total grams carbohydrate to be consumed.</td></tr>
</table>

**Table S2 (cont'd):** Example of instructor using Bloom's taxonomy of educational objectives (cognitive domain) to sequence instruction by complexity of information processing. (Gottfredson, Linda S., and Kathy Stroh. 2021).		
	**4. Analyze**	
	Distinguish, focus, select, integrate, coordinate	Integrate several pieces of information (the two % daily values for carbohydrate) to infer that the label gives carbohydrate grams for milk too, and probably near the column "cereal/w milk".<br>Select correct arithmetic operations to calculate carbohydrate content of each food, plus their total:<br>• X cups of cereal;<br>• The milk for it (note that the label gives no serving size for milk);<br>• Other foods in the meal, labeled or not.
	**5. Evaluate**	
	Check, monitor, detect inconsistencies, judge effectiveness	Evaluate whether:<br>• The intended meal contains the recommended amount of carbohydrate (in grams);<br>• Other circumstances require consuming fewer or more grams of carbohydrate (high or low blood glucose, anticipated physical activity).
	**6. Create**	
	Hypothesize, plan, invent, devise, design	Plan snack or another meal with recommended amount of carbohydrate.<br>Create daily menus with recommended amounts of carbohydrate and other nutrients.
